# Occurrence and Distribution of Tetracycline Antibiotics and Resistance Genes in Longshore Sediments of the Three Gorges Reservoir, China

**DOI:** 10.3389/fmicb.2018.01911

**Published:** 2018-08-17

**Authors:** Lunhui Lu, Jie Liu, Zhe Li, Zhiping Liu, Jinsong Guo, Yan Xiao, Jixiang Yang

**Affiliations:** ^1^CAS Key Laboratory of Reservoir Aquatic Environment, Chongqing Institute of Green and Intelligent Technology, Chinese Academy of Sciences, Chongqing, China; ^2^Key Laboratory of the Three Gorges Reservoir Region’s Eco-Environment, Ministry of Education, Chongqing University, Chongqing, China

**Keywords:** Three Gorges Reservoir, longshore sediments, tetracycline antibiotics, antibiotic resistance genes, spatial-temporal distribution

## Abstract

The widespread use of antibiotics and the induced antibiotic resistance genes have attracted much attention in recent years. The longshore sediments in the water-level-fluctuating zone of the Three Gorges Reservoir were selected to investigate the spatial-temporal distribution of antibiotics and antibiotic resistance genes in two different operation stages (low-water level in summer and high-water level in winter). Three kinds of tetracycline antibiotics (tetracycline, oxytetracycline, and chlortetracycline) and three kinds of tetracycline resistance genes [*tet*(A), *tet*(C), and *tet*(M)] were analyzed and quantified. The results showed that the distribution of tetracyclines and resistance genes in riverine, transition and lacustrine zones showed a certain regularity, and the tetracycline antibiotics concentration and the total abundance of the tetracycline resistance genes were highest in the transition zone, and then the riverine zone. Meanwhile, there were significant seasonal variations of tetracycline and the resistance genes. The concentrations of the tetracycline and resistance genes were higher in summer than those in winter, while the relative abundance of resistance genes was higher in winter. It was suggested that the different seasonal distribution of antibiotics and resistance genes may be correlated with the reservoir operation in the Three Gorges Reservoir and the higher use of antibiotics in winter. In addition, Pearson correlation analysis showed that the concentrations of the tetracycline, class 1 integron and 16S rRNA were positively correlated with the abundance of the tetracycline resistance genes.

## Introduction

In recent years, with the widespread abuse of antibiotics in human medicine and animal husbandry, exogenous antibiotics and antibiotic resistance genes (ARGs) in the environment have become an increasingly global problem (Sarmah et al., 2006). China leads the world in antibiotic production capacity, with a significant percentage of antibiotics utilized in animal agriculture and medicine(Luo et al., 2010). In general, after the consumption, only a small portion of antibiotics can be bio-degraded, most antibiotics are discharged into the environment without being metabolized (Xiong et al., 2018). As the most commonly applied antibiotics, tetracyclines (TCs), such as tetracycline (TC), oxytetracycline (OTC), and chlortetracycline (CTC), are broad-spectrum and affordable (Patterson et al., 2007). Accordingly, TCs are used extensively in medical treatment, as well as for veterinary drugs and growth promoter in poultry farming and aquaculture, particularly in developing countries (Chopra and Roberts, 2001; Roberts, 2003). Remarkably, compared with other antibiotics, TCs are more persistent, highly adsorptive and hardly degradable in soils and sediments (Chee-Sanford et al., 2009). Thus, they are also more easily accumulated (Rabolle and Spliid, 2000; Sarmah et al., 2006; Jiang et al., 2011), posing a potential threat to ecosystems and human health.

TCs may trigger the production of tetracycline resistance genes, which directly cause environmental pollution (Kim, 2004). A typical feature of ARGs is that they can be transferred horizontally between microorganisms using mobile genetic elements as carriers, such as plasmids and transposons (Zhou et al., 2014; Lin et al., 2015). As a type of genetic assembly platform, integrons associated with these mobile genetic elements are considered as the key players in horizontal gene transfer, particularly class 1 integrons (Gaze et al., 2011; Berglund et al., 2014). Through this horizontal gene transfer, ARGs can migrate and transform from different environmental media, and are likely to be introduced into the food chain along with the resistant plasmid and finally enter the human body to increase human antibiotic resistance (Ghosh et al., 2009; Stoll et al., 2012; Sengupta et al., 2013). The aquatic environment is an important pool for ARGs because many pollutants from wastewater treatment plants, industries, hospitals and swine farms finally circulate in water environments and drive the propagation of ARGs (Zhang et al., 2009; Lupo et al., 2012; Yan et al., 2014a,b; Yang et al., 2017). Sediments may act as a sink but also as a secondary source of various contaminants including antibiotics and ARGs (Su et al., 2014), constituting a great potential danger for aquatic organisms (Siedlewicz et al., 2017). Hence, the diversity of antibiotics and ARGs in sediments of the watershed is important for us to understand the diffusion of antibiotic resistance at the catchment scale. To date, reports on the pollution of antibiotics and ARGs in sediments are mainly concentrated in the coastal areas, some rivers and lakes (Pei et al., 2006; Luo et al., 2010; Su et al., 2014; Yang et al., 2016; Calero-Caceres et al., 2017; Siedlewicz et al., 2017). However, there are relatively few studies on longshore sediments in the large reservoir.

As the largest reservoir in China, the Three Gorges Reservoir (TGR) performs several functions, such as flood control, power generation, navigation, irrigation and aquaculture, and its ecological functions are crucial. Since the completion of the Three Gorges Project, the operation of the reservoir has been implemented as an anti-seasonal artificial water level regulation and management method for “winter storage and summer drainage.” The water level rises to 175 m in October and begins to decline in May of the following year. From June to September, the reservoir water level is maintained at the lowest level of 145 m. Periodic changes in reservoir water level affect the sediment deposition in the riparian zone (Wang et al., 2016), which inevitably leads to a change in the transport characteristics of pollutants adsorbed on sediment. Hence, the purpose of this study was to focus on detecting and discussing the spatial-temporal distribution of TCs and tetracycline resistance genes in the longshore sediments of the TGR.

In this study, eight sampling sites were selected in the sediments of the water-level-fluctuating zone of the TGR. Three tetracycline antibiotics (TC, OTC, and CTC) were determined by liquid chromatography-mass spectrometry (LC-MS). Three tetracycline resistance genes [*tet*(A), *tet*(C), and *tet*(M)], one genetic element (class 1 integron, *intI1*) and 16S rRNA gene abundance were assessed by real-time quantitative PCR to reveal the presence of the tetracycline resistance genes in the TGR. This study would give insight into further research on assessment of antibiotics and antibiotic resistance genes pollution in the TGR.

## Materials and Methods

### Site Selection and Sampling

**FIGURE 1:**
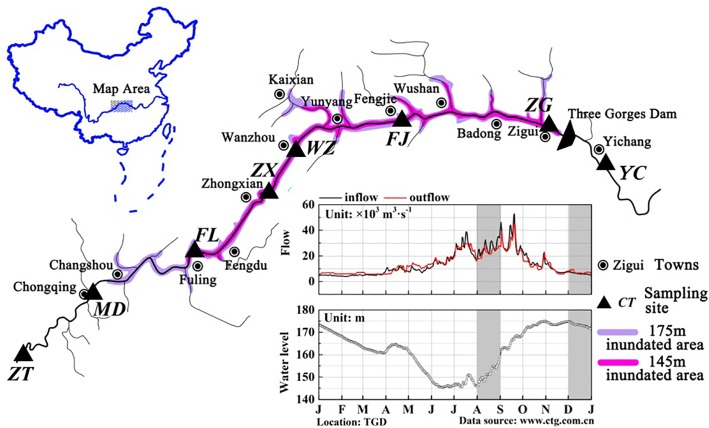
Maps of the TGR showing the location of the sampling sites. Base map of the Yangtze River systems in the TGR and map of China were created by Adobe Photoshop CS5. Sub-graph of daily variations of reservoir in- and outflows, as well as reservoir water levels in 2015, was generated by OriginLab 9.4. Data source was from the web site of the China Three Gorges Cooperation (http://www.ctg.com.cn/). Dark area indicated the time when the samples were taken.

The TGR is formed after the completion of the Three Gorges hydropower station, with a water surface area of 1084 km^2^. This reservoir is from Chongqing (west) to Yichang of Hubei province (east), and the distance is approximately 662.9 km (Chang et al., 2010). According to Thornton et al. (1990), a pure river-like reservoir can be divided into three longitudinal zones: riverine zone, transition zone and lacustrine zone. A riverine zone is located at the upper domain that has a narrow and channelized basin with relatively high flow rates; the lower part immediately upstream of the dam is often described as the lacustrine zone, which has a broad, deep and lake-like basin with low flow rates; a transitional zone is defined between the riverine and lacustrine zones that has a relatively broader and deeper basin with modest flow rates (Kimmel and Groeger, 1984; Thornton et al., 1990). Longitudinal zonation in a reservoir changes in the zone extension due to the difference in hydrodynamic conditions. TGR is periodically regulated by dam operations, and thus exhibits the characteristics of both rivers and lakes, showing the same zonation characteristics. The TGR is a typical reservoir containing three typical zones (riverine zone, transition zone and lacustrine zone) according to the differences in hydrological characteristics, and these three zones are distinguishable along the longitudinal axis (Li et al., 2017). The average water velocity in the sites Zhutuo, Mudong and Fuling (about 0.6∼1.05 m/s in August and 0.30 in December), Fengjie and Zigui (about 0.2 m/s in August and <0.1 m/s in December) (**Supplementary Figure S1**). Moreover, the hydraulic retention time in the TGR varied from 6∼19 days between the sites ZT and FL, from 11∼36 days between Zhongxian and Wanzhou, from 35∼110 days between the sites FJ and ZG. According to the hydraulic retention time and average water velocity calculated, the sampling sites were classified qualitatively. For this reason, eight sampling sites were established along the reservoir: three sites belong to the riverine zone (ZT, MD, and FL), two sites belong to the transition zone (ZX and WZ), two sites belong to the lacustrine zone (FJ and ZG), and one site YC after the dam (**Figure 1**). Detailed information of these sampling sites was summarized in **Table 1**. According to its regulation plan, the water level in the TGR is impounded to 175 m for power generation during the winter and discharged to 145 m for flood control during the summer (Li et al., 2010). Water residence time in August and December represented two periods of the reservoir, i.e., the lotic and lentic periods throughout the year. In this case, sediment samples were collected in the TGR during periods of low-water level (August 2015) and high-water level (December 2015).

**Table 1:** Detailed information about the eight sampling sites.

Sampling sites	Abbreviation	Site characteristics	Location		Distance to the Three Gorges Dam (km)
				
			Latitude (N)	Longitude (E)	
Zhutuo	ZT	Riverine zone	N29°00’47.40″	E105°51’10.20″	778
Mudong	MD		N 29°34’16.20″	E 106°50’22.80″	592
Fuling	FL		N 29°47’31.80″	E 107°27’43.80″	493
Zhongxian	ZX	Transition zone	N 30°25’11.40″	E 108°10’36.60″	352
Wanzhou	WZ		N 30°42’08.40″	E 108°23’15.00″	310
Fengjie	FJ	Lacustrine zone	N 31°02’36.60″	E 109°31’51.00″	164
Zigui	ZG		N 30°51’12.60″	E 110°58’30.00″	2
Yichang	YC	After the dam	N 30°40’31.00″	E 111°18’31.20″	-30


Sampling sites were located along the riparian zone within a distance of 10 m from the water edge, which were subjected to alternate inundation and exposure due to the fluctuation of water level. At each sampling site, three surface sediment subsamples (0–20 cm) were randomly collected by a small shovel. After mixing and splitting into about 1 kg by quartering technique, samples were taken to the laboratory in sterile containers and stored at -80°C for further treatment and analysis (Zhang et al., 2012).

### Physicochemical Properties of the Samples

Sediment samples were air-dried and sieved through a mesh screen (<0.149 mm) and then stored at 4°C before use. Nitrate (NO_3_^-^-N), nitrite (NO_2_^-^-N) and ammonium (NH_4_^+^-N) were extracted from the sediments using 2 M KCl. The extracted NO_3_^-^-N was then determined by a UV spectrophotometry method (Song et al., 2007); the extraction liquid containing NO_2_^-^-N and NH_4_^+^-N was subsequently analyzed according to previously published methods (International Organization for Standardization [ISO], 2003). Water-soluble organic carbon (WSOC) was measured using a Vario TOC Cube analyzer (Elementar, Hanau, Germany). All the above determinations were carried out in triplicate, and the average results were calculated.

### Extraction and Determination of Tetracyclines

Sediment samples were extracted using the most common solid-phase extraction method (Kim and Carlson, 2007a). First, the freeze-dried samples were broken into 0.3 mm particles, and 20 mL McIlvaine buffer was added to 4 g samples. The upper extract of the mixed sample after 30 min of shaking was filtered through a 0.45 μm filter. Then, the filtrate was extracted by an OASIS hydrophilic-lipophilic balance solid-phase extraction column (Waters, Milford, MA, United States), and 5 mL of methanol was added to elute the antibiotics. The eluent was dried by a nitrogen blower, then sealed with 1 mL of methanol and stored at -20°C for LC-MS.

The target compounds TC, OTC and CTC in the extracts were determined by LC-MS. First, analyses were carried out using a Waters ACQUITY UPLC^TM^ system (Waters, Milford, MA, United States). The chromatographic conditions were as follows: chromatographic column, Waters ACQUITY UPLC BEH C18 column (1.7 μm; 2.1 mm × 100 mm); mobile phase, acetonitrile (A) and 0.1% formic acid (formic acid/ultrapure water, V/V) (B); flow rate, 0.2 mL/min; injection volume, 2 μL; column temperature, 30°C. Then, mass spectrometry was performed. The mass spectrometry system consisted of a Waters Micromass Quattro Premier XE (triple-quadrupole) detector and an ion source. The mass spectrometry conditions were as follows: ion source, ESI (+); source temperature, 110°C; desolvation temperature, 400°C; capillary voltage, 2.80 kV; desolvation gas flow, 600 L/h; cone gas flow, 50 L/h. The detailed mass spectrometric operating conditions are found in reference (Jia et al., 2009). For each sample, three replicate assays were performed for LC-MS analysis. The tetracyclines concentrations were all determined based on a dry weight.

### DNA Extraction and PCR

The DNA in the longshore sediment samples was extracted in triplicate with the PowerWater DNA Extraction Kit (Mo Bio, CA, United States) according to the manufacturer’s protocol. The DNA extracts were pooled for each sample to reduce sample variability for the following research. The content and purity of the extracted DNA were detected by 1% (weight/volume) agarose gel and Nanodrop ND-1000 (Nanodrop, United States). The A260/A280 values ranged from 1.8 to 2.0, and A260/230 ranged from 2.2 to 2.5, indicating that the purity of the DNA obtained was high.

Three *tet* genes [*tet*(A), *tet*(C), and *tet*(M)], one genetic element (class 1 integron, *intI1*) and the 16S rRNA gene were investigated. Information about the primers and the annealing temperature is listed in **Table 2**. PCR mixtures contained 25 μL of 2× Power Taq PCR MasterMix (TIANGEN, Beijing, China), 1 μL of each primer (25 μM), and 10 ng of the DNA extracts in a total volume of 50 μL. PCR amplification was run on a MyCycler (Bio-Rad, Hercules, CA, United States) with an initial cycle of denaturation (3 min at 95°C) followed by 30 cycles (30 s at 94°C, 30 s at the annealing temperature (**Table 2**) and 60 s at 72°C), and a final elongation step (5 min at 72°C).

**Table 2:** Information of the PCR primers and amplification conditions.

Target genes	Forward primers	Reverse primers	Amplicon length (bp)	Annealing temperature (°C)	Reference
*tet*(A)	GCTACATCCTGCTTGCCTTC	CATAGATCGCCGTGAAGAGG	210	55	Ng et al., 2001
*tet*(C)	CTTGAGAGCCTTCAACCCAG	ATGGTCGTCATCTACCTGCC	278	55	Aminov et al., 2001
s*tet*(M)	GCAATTCTACTGATTTCTGC	CTGTTTGATTACAATTTCCGC	171	45	Aminov et al., 2001
*intI1*	CCTCCCGCACGATGATC	TCCACGCATCGTCAGGC	280	55	Goldstein et al., 2001
*338F/518R*	CCTACGGGAGGCAGCAG	ATTACCGCGGCTGCTGG	174	55	Koike et al., 2007


The above PCR amplification products were analyzed using electrophoresis with a Bio-Rad Gel Doc2000 on 2% agarose gel electrophoresis. The universal DNA purification and recovery kit (centrifugal column) was then used to purify the sediment DNA in the PCR reaction solution.

### Gene Quantification With Real-time Quantitative PCR

Quantitative PCR (qPCR) was used to quantify the target genes. The 16S rRNA gene was included to quantify the total bacterial load and to normalize the abundance of ARGs (ARG copies/16S rRNA gene copies, defined as relative abundance) in the collected samples. Real-time QPCR was performed on an iCycler iQ5 thermocycler (Bio-Rad, Hercules, CA, United States) to determine the abundance of the target genes in the samples. Standard plasmids carrying target genes were obtained by TA clones and extracted using a TIANpure Mini Plasmid kit (TIANGEN, Beijing, China) (Yu et al., 2005). Concentrations of the standard plasmids (ng/μL) were determined with the Nanodrop ND-1000 spectrophotometer (Nanodrop, United States). Copy concentrations (copies/μL) were then calculated by the following formula (Pei et al., 2006).

$$\text{copy concentration }(\text{copies/g})=\frac{\text{DNA mass concentration }\left(\text{ng/g}\right)}{\text{DNA molecular weight }\left(\text{g/mol}\right)}.$$

The 25 μL qPCR mix contained 2 ng of sediment microbial DNA, 0.75 μL of each primer (25 μM), 10 μL of 2.5× RealMasterMix (TIANGEN, Beijing, China) and 1.5 μL of 20× SYBR solution (TIANGEN, Beijing, China). The protocol was as follows: 15 min at 95°C; 40 cycles consisting of 10 s at 95°C, 20 s at the annealing temperature and 30 s at 72°C; followed by a final extension of 30 s at 72°C. The fluorescent signal was measured at the end of each extension step (Bustin et al., 2009). The melting process was automatically generated by the iCycler iQ5 system.

For the generation of the standards for all qPCR assays, a plasmid construct was used containing the inset of each ARG obtained by conventional PCR. The following requirements were satisfied to obtain reliable quantification (**Supplementary Table S1**): R^2^ was higher than 0.99 for all standard curves over five orders of magnitude. Amplification efficiencies based on slopes were between 95 and 110%. The specificity was assured by the melting curves and gel electrophoresis. The minimum quantification limits for all target genes were within the range of 1.0 × 10^2^ to 1.0 × 10^3^ copies per μL DNA. The qPCR performed included two sets of standards and no-template negative (sterile water) controls on all samples and standards. The presence of PCR inhibitors in the DNA extracted from sediment samples was examined by diluting the DNA extract and mixing a known amount of standard DNA to a DNA extract before qPCR. In none of these cases was inhibition detected. The target genes concentrations were all determined based on a dry weight.

### Statistical Analysis

Pearson correlation analysis was conducted to detect significant differences between physicochemical parameters and the target genes. Differences with *p* < 0.05 were considered statistically significant. The distribution map of antibiotics and resistance genes was completed by Origin 8.0. To understand the distribution characteristics of resistance genes at different sites, principal component analysis (PCA) was performed with Canoco 4.5. Linear-regression analysis was used to assess the association between TCs, *intI1*, 16S rRNA and ARGs. Statistical analyses were performed using SPSS 19.0 (IBM, United States).

## Results

### Physicochemical Parameters of Sediment Samples

The variation of the physicochemical parameters of longshore sediments at the eight sampling sites is presented in **Table 3**. There were significant differences between the parameters at different sites. The mean values of all physicochemical descriptors at site WZ were higher in winter than those in summer. For the other sampling sites, most parameters at the upstream locations of ZT, MD, FL, and ZX appeared analogous distribution characteristics, while only NH_4_^+^-N at the downstream locations of FJ, ZG, and YC were higher in winter than those in summer. In terms of the physicochemical parameters, the mean values of organic matter were higher in summer than in winter. In contrast, WSOC, NH_4_^+^-N, NO_2_^-^-N, and NO_3_^-^-N were higher in winter. Briefly, seasonal differences were noticed between these parameters (*p* > 0.05).

**Table 3:** The physicochemical parameters of the sediments in the TGR.

Time	Sampling site	pH		C^b^ (mS/cm)		OM^c^ (%)		WSOC^d^ (mg/kg)		NH_4_^+^-N (mg/kg)		NO_2_^-^-N (mg/kg)		NO_3_^-^-N (mg/kg)	
								
		Average	SD^a^	Average	SD	Average	SD	Average	SD	Average	SD	Average	SD	Average	SD
2015.8	ZT	7.14	0.05	0.38	0.05	5.46	0.46	9.08	0.66	8.60	0.82	18.56	1.02	3.98	0.70
	MD	7.16	0.06	0.16	0.02	2.93	0.23	7.88	0.60	8.01	0.57	7.72	0.77	11.05	0.86
	FL	6.53	0.25	0.06	0.00	7.82	0.71	8.93	0.71	9.41	0.81	9.27	0.75	3.06	0.23
	ZX	6.58	0.17	0.57	0.06	8.53	0.70	10.59	0.42	8.12	0.53	15.34	0.94	9.53	0.31
	WZ	6.88	0.11	0.22	0.02	4.79	0.52	13.90	1.45	8.21	0.28	7.41	0.59	1.43	0.15
	FJ	6.40	0.31	0.74	0.05	11.22	1.05	19.38	1.67	7.61	0.62	10.21	0.83	25.50	2.29
	ZG	7.56	0.27	0.74	0.04	6.84	0.72	17.70	1.11	7.69	0.58	13.05	1.02	14.04	0.54
	YC	7.46	0.17	0.11	0.04	5.11	0.43	26.20	1.90	5.95	0.22	10.54	0.93	8.24	0.66
2015.12	ZT	7.24	0.36	0.89	0.10	2.89	0.27	17.86	1.27	10.67	0.63	12.51	0.81	23.24	0.48
	MD	6.64	0.25	0.48	0.08	3.79	0.59	22.40	2.26	9.37	0.53	11.22	0.84	5.45	0.45
	FL	6.85	0.28	0.41	0.04	4.51	0.49	14.16	1.81	8.56	0.50	16.30	0.87	5.12	0.44
	ZX	6.96	0.00	0.26	0.05	10.75	0.98	36.24	2.34	10.72	0.57	22.51	1.09	23.07	0.55
	WZ	7.07	0.32	0.39	0.04	5.54	0.44	17.79	1.96	11.18	0.96	14.34	0.60	10.03	0.57
	FJ	6.11	0.22	0.22	0.03	6.81	0.45	25.59	1.58	9.84	0.64	19.75	1.13	16.17	0.48
	ZG	6.59	0.18	0.22	0.03	4.15	0.28	12.41	1.42	8.05	0.82	8.27	0.72	3.31	0.30
	YC	6.52	0.17	0.18	0.02	3.32	0.28	21.89	1.80	8.24	0.50	10.40	0.82	6.29	0.49


*Abbreviations: a, standard deviation; b, conductivity; c, organic matter; d, water-soluble organic carbon.*

Pearson correlation analysis of these indicators at each site showed that the conductivity and WSOC were significantly correlated with NO_3_^-^-N, with *p* values of 0.023 and 0.040, respectively, while there was no obvious correlation between other physicochemical indicators (**Supplementary Table S2**).

### Spatial-Temporal Distributions of Tetracyclines

**FIGURE 2:**
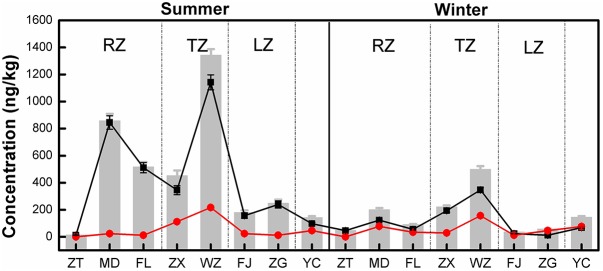
Distribution of tetracycline antibiotics in longshore sediments in the TGR. Black and red symbols represented the mean concentrations of TC and OTC, respectively, and the gray bars indicated the total concentration of tetracycline antibiotics at each site (the total concentration was the sum of the mean concentrations of TC and OTC). RZ, TZ, and LZ respectively represented the riverine zone, transition zone and lacustrine zone of the TGR. Error bars represent the standard deviations.

Of the three tetracycline antibiotic compounds, CTC was only detected at Wanzhou site in the summer. TC and OTC were detected at all sampling sites in both sampling seasons. The results of the concentrations of all detected tetracyclines are shown in **Figure 2**. The TC concentration ranged from 12.11 to 1142.67 ng/kg in the summer, 11.74 to 347.21 ng/kg in the winter. For OTC, the concentration ranged from 0.33 to 216.93 ng/kg in the summer and 0.12 to 156.44 ng/kg in the winter. In addition, the mean concentrations of TC and OTC were 263.60 and 54.73 ng/kg, respectively. Clearly, TC was the dominant tetracycline antibiotic in the TGR, contributing 20.45–99.74% to the total tetracycline antibiotic detected. Except for ZT and TC concentrations at all sites were noticeably higher in the summer. For OTC, only the concentrations at ZT, ZX, WZ, and FJ were slightly higher in summer, indicating that the distribution of OTC did not have a clear seasonal variation. On the other hand, the concentrations of TC and OTC in WZ were the highest, followed by MD and FL. The concentrations of TC and OTC were added together as the sum of the concentrations of tetracyclines at each site, and recorded as TCs (the gray bars in **Figure 2**). It was found that the concentration of TCs in the transition zone (ZX and WZ) was the highest, followed by the riverine zone (ZT, MD, and FL), and the lacustrine zone (FJ and ZG) was the lowest.

### Distribution of Tetracycline Resistance Genes

**FIGURE 3:**
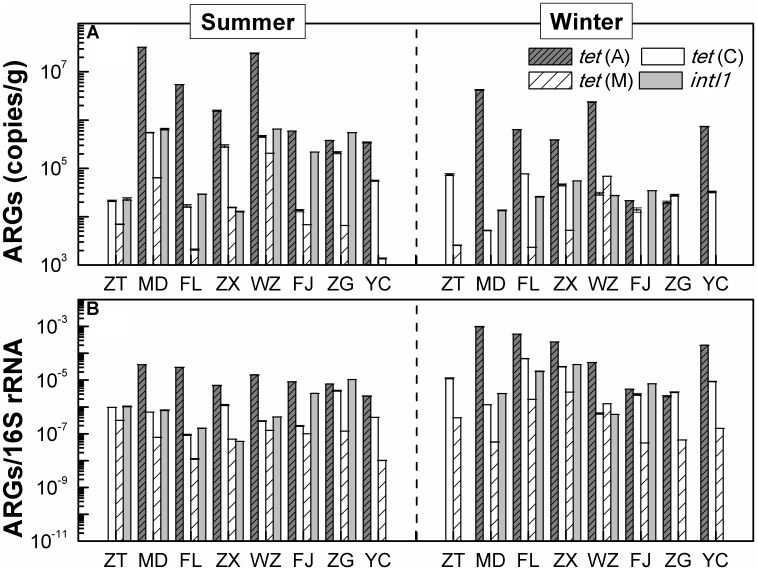
The distribution of ARGs. **(A)** The absolute abundance distribution of ARGs. **(B)** The distribution of ARGs/16S rRNA (relative abundance of ARGs), copies/16S gene copies. Each value is the mean ± SD of three replicates.

**Figure 3** shows the occurrences and quantities of ARGs and *intI1* in the sediments of the TGR. Of tetracycline resistance genes analyzed, *tet*(C) and *tet*(M) had 100% detection frequency in all eight sediment samples of the TGR, followed by *tet*(A) (87.5%). The concentrations of these three genes varied greatly by five orders of magnitude, ranging from 2.11 × 10^2^ copies/g [*tet*(M) of site MD in the winter] to 3.23 × 10^7^ copies/g [*tet*(A) of site MD in the summer]. Additionally, among the three investigated genes, the absolute and relative abundance of *tet*(A) was the highest, while the *tet*(M) gene was found with the lowest average absolute abundance and relative abundance. *IntI1* was also detected in sediment samples; however, the detection frequency (75%) was lower than that of *tet* genes, with mean absolute abundance of 1.91 × 10^5^ copies/g and mean relative abundance of 7.14 × 10^5^ copies/16S rRNA. On the other hand, from the time perspective, the absolute concentration of ARGs and *intI1* in summer was higher than that in winter, while the relative abundance of ARGs and *intI1* was the opposite.

**FIGURE 4:**
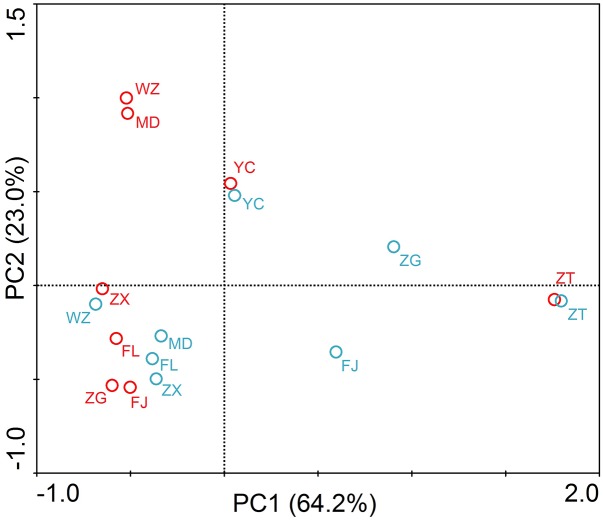
Principle component analysis (PCA) of absolute concentrations of three *tet* genes, 16S rRNA and *intI1* in the TGR. The circle in the graph represented the sampling site. Red circle and blue circle represented the samples in summer and winter, respectively. The PC1 and PC2 explained 64.2 and 23.0% of the total variance, respectively. Seven sampling sites along the longitudinal axis in the TGR were selected from upstream to downstream: riverine zone consisted of sites ZT, MD, and FL; the transition zone consisted of sites ZX and WZ; the lacustrine zone consisted of FJ and ZG. YC belonged to the site after the dam.

The principle component analysis (PCA) was carried out to reveal the distribution characteristics of the target genes at different sites (**Figure 4**). As shown in the figure, ZT was located at the end of the PCA graphs. Principal components 1 and 2 accounted for 87.2% of the total sample variability (64.2 and 23.0% for PC1 and PC2, respectively). PCA coordinates showed comparatively high discrepancies between the different sites and periods in the TGR. In summer, WZ and MD had a highly similar distribution of resistance genes, and it was probably due to the sites affected by human activities seriously. Sites of the lacustrine zone (FJ and ZG) clustered together in the PCA plot, showing a certain similarity. However, the riverine and transition zone did not show similarity between the sites in the same zone. In winter, the two sites of the riverine zone (MD and FL) and the two sites of the transition zone (ZX and WZ) were clustered together.

### Distribution of TCs and ARGs in Relation to Environmental Variables Within the TGR

To explore the factors influencing the distribution characteristics of TCs and ARGs, some environmental factors (physicochemical indicators detected in this study) and social factors (population density, GDP per capita, GDP, land area, and permanent population^1^) were selected for Pearson correlation analysis. In summer, no correlation was found between any factors (**Supplementary Table S3**). But in winter, there was a significant correlation between *intI1* and organic matter, NH_4_^+^-N, NO_2_^-^-N, and NO_3_^-^-N (**Supplementary Table S4**). Besides, an obvious correlation was found between *intI1* and permanent population, land area, population density, organic matter, which indicating that *intI1* was greatly affected by these factors in winter. When all data in both seasons were grouped together, as shown in **Supplementary Table S5**, correlation analysis demonstrated that only *tet*(A) had negative correlation relationship with NO_2_^-^-N (*p* = 0.047).

### Correlation Between ARGs Abundances and Antibiotic Concentrations

**FIGURE 5:**
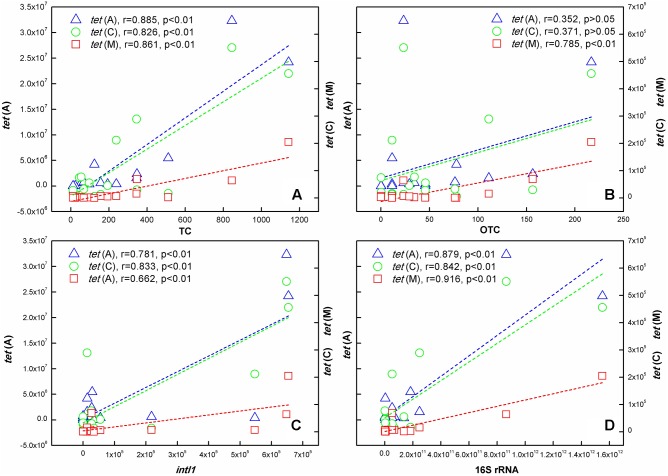
Correlation analysis identified the relationship of 16S rRNA, *intI1*, TCs, and ARGs in samples collected in the TGR. **(A)** TC; **(B)** OTC; **(C)** *intI1*; **(D)** 16S rRNA. [The left ordinate indicated the content of *tet*(A); the right ordinate indicated *tet*(C) and *tet*(M) content].

As shown in **Figure 5**, significant correlations existed between TC concentration and the three *tet* genes abundance at the *p* < 0.01 level. The correlation between OTC concentration and tetracycline resistance genes was different. It was noteworthy that no correlation was found between *tet*(A), *tet*(C) abundance and OTC concentration, but there was a significant correlation between *tet*(M) abundance and OTC concentration. In addition, Pearson correlation analysis also showed that *intI1* and 16S rRNA abundance had positive correlation relationships with the three *tet* genes abundance at the *p* < 0.01 level.

## Discussion

### Relationships Between TCs, *intI1*, 16S rRNA, and ARGs

*Tet* genes can be classified into four categories according to their different resistance mechanisms: efflux genes, ribosomal protection genes, enzymatic genes and other genes (Roberts, 2005). The three *tet* genes detected in this study cover two of them: the efflux genes [*tet*(A) and *tet*(C)] and the ribosome protection genes [*tet*(M)]. Previous studies had shown that efflux genes were more common in the environment compared with ribosomal protection genes (Borjesson et al., 2010). This may be the reason why the abundance (absolute and relative) of *tet*(M) was quite different from that of *tet*(A) and *tet*(C). Chopra and Roberts (2001) pointed out that ribosomal protection protein conferred a wider spectrum of resistance to TCs than those carrying tetracycline efflux proteins, with the exception of *tet*(B). Correlation analysis with antibiotics in this study showed that *tet*(A) and *tet*(C) had a relatively consistent correlation, while *tet*(M) was opposite. This difference may be caused by different mechanisms of the genes. However, due to the limited number of genes selected in this study, the above phenomenon may be accidental, and further research is needed to verify it.

Apart from the possible internal factors of the resistance mechanism, many external factors were also the key factors affecting the distribution of antibiotics and resistance genes, like environmental conditions (Siedlewicz et al., 2017) and human activities (Pruden et al., 2006; Zhou et al., 2011; Su et al., 2014; Yang et al., 2016). However, the sampling location and sampling strategy selected by different research institutes were inconsistent, which may be the reason for the lack of correlation between antibiotics, ARGs and other factors chosen in this study. Except for the above elements, other factors also affected the abundance of resistance genes, such as antibiotics (Luo et al., 2010), *intI1* (Zhu et al., 2013) and 16S rRNA (Chen and Zhang, 2013). In theory, as the direct selection pressure of ARGs, the abundance of ARGs in the environment was consistent with the corresponding concentration of induced antibiotics (Wang et al., 2014; Niu et al., 2016). However, some articles suggested that there was no correlation between some antibiotics and their corresponding ARGs (Niu et al., 2016). Because in addition to the above factors, light (Pei et al., 2007), temperature (Engemann et al., 2008), heavy metals (Baker-Austin et al., 2006; Gillings and Stokes, 2012) and other factors also affect the abundance of ARGs, especially antibiotic resistance can be co-selected by heavy metals (Zhu et al., 2013). Nevertheless, antibiotic residues are still considered to be the direct and primary selection pressures for acceleration of the expansion of the bacterial resistance (Caucci et al., 2016). In generally, such a significant difference existed in the association of antibiotics with ARGs, one of which may be due to the different habits of antibiotic use in different regions and the different chemical properties of different ARGs. *IntI1* was known to play a key role in horizontal gene transfer, leading to widespread antibiotic resistance (Zhu et al., 2013). Meanwhile, *intI1* was also regarded as an environmental marker of anthropogenic pollution (Gillings et al., 2015). The high correlation between the *tet* genes and *intI1* revealed the potential horizontal gene transfer in these samples. The characteristics of the bacterial abundance were analyzed based on 16S rRNA. As the potential hosts of ARGs, bacteria would amplify or attenuate under different conditions, so the persistence and proliferation of ARGs were also closely associated with the bacterial abundance (Su et al., 2017). The above results indicated that the higher abundance of bacteria in sediments in summer than that in winter may play an important role in the horizontal spread of *tet*(A), *tet*(C) and *tet*(M) genes in the TGR.

### Spatial-Temporal Variation of TCs and ARGs in the Sediments of the TGR

The TGR is different from other basins, with a low-water level in summer and a high-water level in winter. The seasonal distribution of TGR antibiotics and resistance genes was also different from other basins that was, the concentration of TC and the abundance of ARGs were higher in summer than those in winter. In general, many factors would cause antibiotics to present different distribution characteristics in different seasons, such as temperature and water flow rate (Kim and Carlson, 2007b; Loftin et al., 2008). For example, photo degradation and biodegradation were less effective at low temperature in winter (Cheng et al., 2014). At the same time, TCs were commonly administered to livestock for the prevention and treatment of most respiratory infection and diarrhea (Matsui et al., 2008) and their usage tended to increase in winter (Cheng et al., 2014). Under the influence of these factors, most studies found that the concentration of antibiotics in the water body was higher in the dry season (Yan et al., 2013; Chen et al., 2018). The seasonal variation in antibiotics would produce a selective pressure, so the abundance of ARG in winter would be higher (Caucci et al., 2016; Sui et al., 2017). But the opposite phenomenon were also found in other literatures (Arikan et al., 2008; Jiang et al., 2011; Knapp et al., 2012; Calero-Caceres et al., 2017), which indicating that the seasonal distribution of antibiotics and ARGs was controversial. It was suggested that the hydrological conditions of different basins may be inconsistent, therefore we speculate that the seasonal distribution in this study may be caused by unique hydrological conditions of the TGR.

It had been reported that the sediment could act as a significant secondary source of antibiotics and could release pollutants into the water if the aquatic environment changed (Cheng et al., 2014), with the antibiotics strongly bonding in the sediments. This tended to protect them from potential degradation processes, which resulted in a greater persistence and a longer-term fate of the antibiotics (Chen and Zhou, 2014). In summer, the TGR is no longer at the high-water level, but operates stably at the low-water level of 145 m. The aquatic environment was changed, sediments in the riparian zone were also changed, and fluctuation of the water level turned the original bottom sediment in winter into the longshore sediment in summer. Previously, the antibiotics in water bodies were adsorbed and accumulated in sediments by natural sedimentation, which resulting in an increase in the concentration of TCs in these sediments. On the other hand, heavy rainfall occurred in the summer, and rainfall runoff carried a large amount of antibiotics into the water, leading to an increase in external input. As the main selection pressure of ARGs, the concentration of TCs in the water during the summer was higher and thus the absolute abundance of ARGs was also higher. In addition, as the rainfall increased, the velocity of the flowing water in the reservoir also increased. ARGs could migrate among different environmental compartments (Chen et al., 2013). Hence, it was reasonable to assume that aqueous-phase mechanical agitation should be enhanced, possibly resulting in the increased exchange of ARGs between the longshore sediments and the water phase (Fang et al., 2017), thereby resulting in a higher concentration of ARGs in the longshore sediments. Meanwhile, we also hypothesized that more frequent exchanges of ARGs may likely occur in the summer than in the winter, bringing about higher concentrations of ARGs in the sediments. This was maybe because the warmer temperatures may promote the survival of bacteria in sediments, and thus lead to a greater number of copies of ARGs (Luo et al., 2010). This assumption was supported by our findings that the abundance of 16S rRNA in sediments was higher in the summer than in the winter and that massive microbial carriers of the ARGs provided the fundamental conditions for the exchange of ARGs (Fang et al., 2017). In December, the reservoir operated at a high-water level and the water flow rate slowed down, which in turn accelerated the sedimentation of antibiotics in the water, leading to a lower concentration of TCs and ARGs in the longshore sediments.

The distribution of TCs and ARGs exhibited clear spatial heterogeneity. Reservoirs are created ecosystems that behave as “hybrids” of river and lake ecosystems (Scott et al., 2009). The riverine, transition and lacustrine zones are three typical zones in the TGR. These longitudinal gradients influence the physical, chemical and biological processes that affect sedimentation (Liu et al., 2012). Along the longitudinal axis, sites MD, FL, and WZ were near a large metropolitan area with a large population and sizeable industries (Li et al., 2017), which resulted in more external antibiotics and ARGs flowing into the TGR. The sediments in the riverine zone were mainly exogenous (Cooke et al., 2011), so the concentrations of antibiotics and resistance genes in the riverine zone were relatively high. However, the riverine zone was close to the inlet of the reservoir, where the fastest water flow rate led to a degree of dilution of the antibiotics and resistance genes. The sediments in the transition zone were exogenous and endogenous (Filstrup and Lind, 2010). Due to the large input of WZ, the content of antibiotics and ARGs in the transition zone were also highest among the eight sites in this study. Moreover, and the decrease in the reservoir flow rate in the transition zone resulted in the residence of the antibiotics and ARGs. Besides, it was worth noting that, in addition to ZT, the concentrations of antibiotics and ARGs at upstream sites (ZT, MD, FL, ZX, and WZ) were higher than those at downstream sites (ZG and FJ), in agreement with previous studies on the Liao River (Dong et al., 2016). Our previous work indicated that anthropogenic activities might lead to the different characteristics of the upstream and downstream sites (Li et al., 2017). Hence, fewer exogenous inputs at FJ and ZG may also result in lower concentrations of antibiotics and ARGs at the downstream sites. In addition, the relatively low antibiotic concentration at the downstream sites could be mainly attributed to dilution of the water from the TGR (Loftin et al., 2008).

## Conclusion

This study demonstrated the occurrence and distribution of antibiotic and ARGs in the longshore sediments of the TGR, China. The differences in hydrological conditions and surrounding environmental conditions also probably led to different distribution characteristics of antibiotic and resistance genes in three typical zones of the TGR (riverine, transition, and lacustrine zones). Statistical analyses revealed that both antibiotics and 16S rRNA played a role in the distribution of ARGs within the TGR. Besides, the concentration of *tet* genes was significantly correlated with *intI1* (*p* < 0.05) implying horizontal transfer of ARGs, which posed a great threat to public health. It is necessary to conduct further researches on the environmental effects and migration transformation mechanisms of the ARGs.

## Author Contributions

LL, ZL, ZPL, and JG conceived and designed the study. JL, YX, and JY performed the experiments. LL, ZL, JL, ZPL, and JG analyzed the data and wrote the paper. All authors contributed to the editing of the manuscript.

## Conflict of Interest Statement

The authors declare that the research was conducted in the absence of any commercial or financial relationships that could be construed as a potential conflict of interest.

## Footnotes

1 http://www.cqtj.gov.cn/
