# Supplementary material for: Occurrence and Distribution of Tetracycline Antibiotics and Resistance Genes in Longshore Sediments of the Three Gorges Reservoir, China
Source: Front Microbiol. 2018 Aug 17;9:1911. doi: 10.3389/fmicb.2018.01911 (PMC6108234; doi:10.3389/fmicb.2018.01911)
Supplement: Supplementary file 1 [file Image_1.pdf]

Supplementary Material

Occurrence and Distribution of Tetracycline Antibiotics and Resistance Genes in Longshore Sediments of the Three Gorges Reservoir, China

Lunhui Lu[1#], Jie Liu[2#], Zhe Li[1,2*], Zhiping Liu[2*],Jinsong Guo[2], Yan Xiao[1], Jixiang Yang[1]

[#] These two authors contributed equally to this work and share the first authorship.

[1] CAS Key Laboratory of Reservoir Aquatic Environment, Chongqing Institute of Green and Intelligent Technology, Chinese Academy of Sciences, Chongqing, China

[2] Key Laboratory of the Three Gorges Reservoir Region's Eco-Environment, Ministry of Education, Chongqing University, Chongqing, China

[*] Correspondence:

Zhe Li

lizhe@cigit.ac.cn

Zhiping Liu

liulqs@163.com

Supplementary Tables

Table S1. Quality control of the real-time PCR methods for all the target genes.

Genes	Quantification limit of method (copies/ µL DNA)	R^2	Slope	Efficiency* (%)
tet(A)	1.0×10^2	0.9995	-3.36	98.44
tet(C)	1.0×10^2	0.9994	-3.38	97.54
tet(M)	1.0×10^2	0.9997	-3.39	97.19
intI1	1.0×10^2	0.9987	-3.39	97.16
16S rRNA	1.0×10^3	0.9981	-3.24	103.74

*: Efficiency = $(10^{1/slope}-1)\times100$ (%).

Table S2. Correlations between the physicochemical parameters of the samples in the TGR[#].

	**pH**	**Conductivity**	**Organic matter**	**WSOC**	**NH_4^+-N**	**NO_2^--N**	**NO_3^--N**
pH	1						
Conductivity	0.205	1					
Organic matter	-0.276	0.154	1				
WSOC	-0.026	-0.018	0.317	1			
NH_4^+-N	-0.168	0.144	0.057	0.220	1		
NO_2^--N	-0.043	0.122	0.418	0.464	0.473	1	
NO_3^--N	0.031	**0.562**[*]	0.495	**0.517**[*]	0.290	0.359	1

[#] Values indicated the Pearson correlation coefficient (r).

[*] Correlation is significant at the 0.05 level (2-tailed).

[**] Correlation is significant at the 0.01 level (2-tailed).

Table S3. Correlations between the influencing parameters and TCs, ARGs in the summer[#].

	**TC**	**OTC**	***tet*(A)**	***tet*(C)**	***tet*(M)**	***intI1***	**16S rRNA**
PP[a]	-0.125	0.127	-0.074	-0.194	0.018	-0.305	0.036
GDP	-0.165	0.035	-0.105	-0.226	-0.067	-0.328	-0.035
LA[b]	-0.305	-0.011	-0.253	-0.301	-0.178	-0.344	-0.166
GDP per capita	0.123	-0.052	0.187	-0.111	0.034	-0.251	0.127
PD[c]	0.299	0.065	0.446	0.267	0.315	0.060	0.346
pH	-0.151	-0.201	0.036	0.156	-0.047	0.285	-0.044
Conductivity	-0.422	-0.131	-0.470	-0.161	-0.274	0.066	-0.396
OM[d]	-0.450	-0.151	-0.648	-0.581	-0.428	-0.436	-0.524
WSOC	-0.388	0.010	-0.414	-0.353	-0.156	-0.118	-0.236
NH_4^+-N	0.312	-0.018	0.175	0.046	0.125	0.025	0.125
NO_2^--N	-0.705	-0.316	-0.660	-0.429	-0.512	-0.545	-0.617
NO_3^--N	-0.382	-0.367	-0.256	-0.183	-0.395	0.071	-0.397

[#] Values indicated the Pearson correlation coefficient (r).

[*] Correlation is significant at the 0.05 level (2-tailed).

[**] Correlation is significant at the 0.01 level (2-tailed).

Abbreviations: a, Permanent population; b, Land area; c, Population density; d, Organic matter.

Table S4. Correlations between the influencing parameters and TCs, ARGs in the winter[#].

	**TC**	**OTC**	***tet*(A)**	***tet*(C)**	***tet*(M)**	***intI1***	**16S rRNA**
PP[a]	0.067	0.356	0.052	-0.018	0.073	-0.428	0.062
GDP	-0.018	0.306	0.036	0.014	-0.017	-0.488	-0.026
LA[b]	-0.124	0.209	-0.106	-0.124	-0.112	-0.477	-0.107
GDP per capita	0.035	0.316	0.289	0.306	0.029	-0.429	-0.010
PD[c]	0.293	0.041	0.401	0.387	0.226	0.243	0.204
pH	0.473	0.155	0.064	0.657	0.406	0.281	0.344
Conductivity	-0.010	-0.216	0.109	0.521	0.036	0.102	0.051
OM[d]	0.372	-0.111	-0.185	-0.094	0.098	**0.914****[**]**	-0.016
WSOC	0.255	-0.180	0.003	-0.228	-0.136	0.712	-0.238
NH_4^+-N	0.667	0.151	0.131	0.086	0.569	**0.766****[*]**	0.511
NO_2^--N	0.245	-0.285	-0.241	0.142	0.040	**0.965****[**]**	-0.093
NO_3^--N	0.116	-0.469	-0.387	0.320	-0.029	**0.889****[**]**	-0.089

[#] Values indicated the Pearson correlation coefficient (r).

[*] Correlation is significant at the 0.05 level (2-tailed).

[**] Correlation is significant at the 0.01 level (2-tailed).

Abbreviations: a, Permanent population; b, Land area; c, Population density; d, Organic matter.

Table S5. Correlations between the influencing parameters and TCs, ARGs[#].

	**TC**	**OTC**	***tet*(A)**	***tet*(C)**	***tet*(M)**	***intI1***	**16S rRNA**
PP[a]	-0.062	0.215	-0.044	-0.119	0.028	-0.210	0.024
GDP	-0.101	0.141	-0.065	-0.136	-0.047	-0.227	-0.022
LA[b]	-0.201	0.076	-0.172	-0.193	-0.139	-0.248	-0.106
GDP per capita	0.079	0.095	0.143	-0.045	0.028	-0.166	0.079
PD[c]	0.226	0.054	0.320	0.191	0.252	0.122	0.220
pH	0.112	-0.068	0.135	0.281	0.121	0.384	0.107
Conductivity	-0.280	-0.161	-0.330	-0.079	-0.189	0.134	-0.275
OM[d]	-0.061	-0.124	-0.315	-0.212	-0.170	-0.131	-0.189
WSOC	-0.375	-0.069	-0.396	-0.422	-0.239	-0.277	-0.341
NH_4^+-N	-0.080	0.036	-0.149	-0.283	-0.009	-0.262	-0.226
NO_2^--N	-0.468	-0.276	**-.0503***	-0.372	-0.358	-0.418	-0.479
NO_3^--N	-0.262	-0.398	-0.240	-0.149	-0.288	0.065	-0.302

[#] Values indicated the Pearson correlation coefficient (r).

[*] Correlation is significant at the 0.05 level (2-tailed).

[**] Correlation is significant at the 0.01 level (2-tailed).

Abbreviations: a, Permanent population; b, Land area; c, Population density; d, Organic matter.

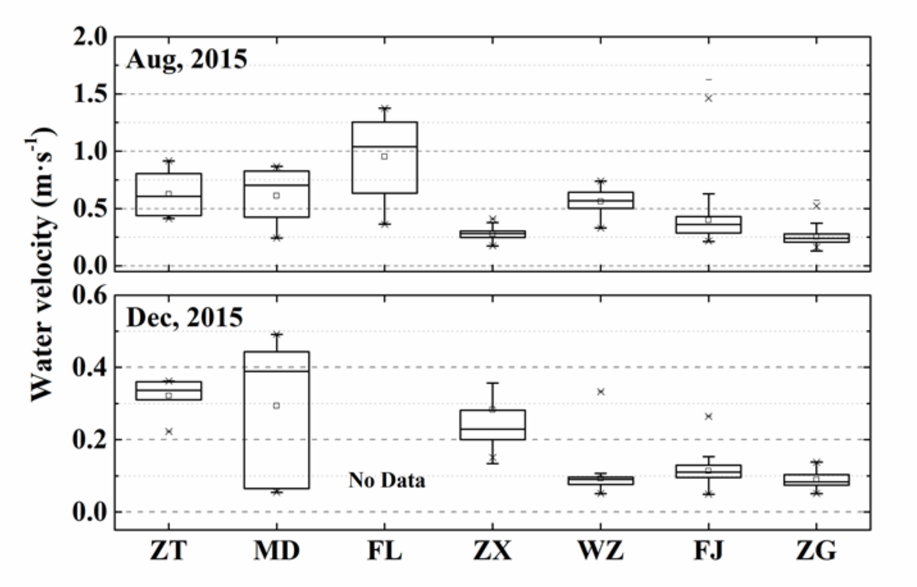

Figure S1 The average water velocity of the sampling sites in August and December.
